# Insights into controlling role of substitution mutation, E315G on thermostability of a lipase cloned from metagenome of hot spring soil

**DOI:** 10.1007/s13205-013-0142-4

**Published:** 2013-06-02

**Authors:** Pushpender Kumar Sharma, Rajender Kumar, Prabha Garg, Jagdeep Kaur

**Affiliations:** 1 Department of Biotechnology, Panjab University, Sector 14, Chandigarh, 160014 India; 2 Department of Biotechnology, Sri Guru Granth Sahib World University, Fatehgarah Sahib, India; 3 Department of Pharmacoinformatics, National Institute of Pharmaceutical Education and Research (NIPER), S.A.S. Nagar, Mohali, 160062 Punjab India; 4 Computer Centre, National Institute of Pharmaceutical Education and Research (NIPER), S.A.S. Nagar, Mohali, 160062 Punjab India

**Keywords:** Lipase, Mutagenesis, Thermostability, Conformation, α/β hydrolase fold, Homology modeling

## Abstract

Rational mutagenesis was performed (at the vicinity of the active site residues D317 and H358 of a mature polypeptide) to investigate the role of amino acids in the thermostability/activity of a lipase enzyme. The single variant enzyme created with E315G (lip M2) mutation near one of the active site residue (D317) found to be an important residue in controlling the thermal stability, the variant with E315G mutation demonstrated biochemical properties similar to that of native lipase. However, we found that this mutation strongly affected the activity and stability of the lip M1 mutant, reported in our previous study (Sharma et al. in Gene 491:264–271, 2012b). The dual mutant with E315G/N355K mutation in the Wt showed small increase in the protein thermostability compared to the native lipase, however, the thermostability of the mutant lip M1 was reduced several fold. Presumably, E315G (lip M2) mutation reverted the thermostability evolved by N355K (lip M1). The native and variant enzymes also displayed large variation in enzyme kinetics and their preference for pNP-esters (substrates). We further generated 3D models and studied the loop modelling of the WT and variants. Interestingly, loop region Leu314-Asn321 showed structural flexibility on introducing E315G mutation in the native lipase. On the other hand, lysine in mutant N355K exhibited side chain conformational changes in the loop Thr353-His358 which resulted in its H-bonding with Glu284. In addition, replacing glutamic acid by glycine at 315 position in lip M3 distorted the electrostatic interactions between Glu315 and Lys355 in the flexible loop region Leu314-Asn321.

**Electronic supplementary material:**

The online version of this article (doi:10.1007/s13205-013-0142-4) contains supplementary material, which is available to authorized users.

## Introduction

Recent advances in genetic engineering methods offers valuable tools to modify enzyme activity with desired functions (Kazlauskas and Bornscheuer 2009; Lutz and Bornscheuer 2009). Despite these advances, the intrinsic properties of the enzymes are difficult to explain or predict, as even slight change in the altered structure can affect both enzyme activity and stability (Bloom et al. 2006; Spiller et al. 1999; Shimotohno et al. 2001). Therefore, understanding structure function relation at molecular level is multifaceted and raised many question i.e. why certain protein catalyzes a biochemical reaction more efficiently, and why certain proteins are more thermostable than the other (Bouzas et al. 2006). In the past several years, many researchers have employed a variety of molecular tools to improve the catalytic function of the enzymes (Magnusson et al. 2005; Reetz 2000; Koga et al. 2003). Typically, the factors which are considered important in enhancing protein thermostability are increased hydrophobicity, rigidity, compactness in the structure, decrease percentage age of thermolabile residues, increased hydrogen bonding and presence of salt bridge etc. (Haney et al. 1997; Sadeghi et al. 2006; Russel et al. 1997; Bogin et al. 1998; Gromiha 2001; Kumar et al. 2000), whilst, reverse may be true for less thermostable proteins. It is also suggested that the energy difference between a stable versus unstable proteins is very small (Tokuriki and Tawfik 2009). In addition, it was also stated that thermostability of enzyme is related to the rigidity of a protein structure and can affect protein function to much more extent. In this particular case, we are studying the effect of substitution mutation on the activity and stability of a lipase that had shared more than 90 % homology with thermostable enzyme of *Bacillus* species. Lipases belong to a group of enzyme that catalyzes the synthesis and hydrolysis of long chain of fatty acids. These are important biocatalysts and find numerous applications in different industries that mainly include food, leather, pharmaceutical, dairy and detergent making industry (Jaeger et al. 1999). They belong to serine proteases and include a conserved Gly-X-Ser-X-Gly motif, near the catalytic residue serine (Kim et al. 1998). In addition to serine, their catalytic triad contains aspartate, and histidine (Choi et al. 2005). Structurally, lipase belongs to α/β hydrolase fold that comprises parallel β-strands surrounded by α-helices (Schrag and Cygler 1997; Cherukuvada et al. 2005). These enzymes had been modified previously for enhancing protein thermostability, catalytic function, and for the development of enantiomeric pure compounds etc. Recent studies have shown that the stabilizing mutation which conferred thermostability had not resulted in loss of the structural rigidity in lipase mutants derived from the native lipases of a mesophilic *Bacillus* sp. (Acharya et al. 2004; Ahmad et al. 2008). Previously, we reported cloning and characterization of a gene encoding extracellular lipase in detail (Sharma et al. 2012a, b). Recently, we described a highly thermostable mutant lip M1, carrying mutation N355K close to the active site of the WT enzyme (Sharma et al. 2012a). Interestingly, during multiple sequence alignment, we notice that lysine at 355 position was critical in determining protein thermostability and was conserved in homologous proteins. Furthermore, we observed another alteration in amino acid sequence of this polypeptide mutation close to aspartate (a catalytic residue at 317 position), where the conserved glycine at 315 position was replaced to glutamic acid. Therefore, we set out our objective to mutate glutamic acid to the conserved glycine, in both native and N355K. All purified enzymes (WT and variants) characterized biochemically for various properties demonstrated great variations (Table 1). We, next performed molecular dynamics in the loop region to shed light into structural plasticity at three dimensional levels.

## Materials and methods

### Reagents/kits/plasmids

pGEM-T easy vector (Promega, USA) was used for cloning and pQE30-UA plasmid (Quiagen, Germany) was used for expression purpose. Gel extraction kit was purchased from MOBIO (USA). Taq DNA polymerase (5 U/μl), dNTPs mix, each was purchased from Fermentas (Germany). Substrates (pNP-esters and tributyrin), used for biochemical assays and screening, were purchased from Sigma Aldrich (USA). All other chemicals were procured from Merck (Germany). The WT and lip M1 plasmid DNA with mutation (N355K) used in this study were cloned earlier in the lab.

### Site specific mutagenesis and molecular manipulations

Site directed mutagenesis was carried out by overlap extension PCR. The full length primer used were as follows: 5′-TGATGAARGGNTGYAGRGTNCC-3′ (forward) and 5′-TTANGGNCGNA (A/G) N(C/G) (T/A) NGCNA (G/A) (T/C) TGNCC-3′ (reverse). Primer sequences used for the amplification of single mutant encoding E315G were as follows: 5′-ATTGGCTTG**G**GAACGACGG-3′ (forward) and 5′-CCGTCGTTC**C**CAAGCCAAT 3′ (reverse). Double mutant encoding E315G/N355K was generated using the plasmid DNA extracted from clone E315G. Oligonucelotide sequences used for amplification mutant encoding N355K were as follows: 5′-TGG AAT GAC ATG GGA ACG TAC AA**G**GTC GAC CAT TTG G 3′ (forward) and 5′-CCA AAT GGT CGA C**C**T TGT ACG TTC CCA TGT CAT TCC A-3′ (reverse). PCR (gradient) reaction was performed in a Bio-Rad thermal cycler as follows: 94 °C for 4 min, followed by 30 cycles at 94 °C for 1 min, 55/59.5 °C for 50 s and 72 °C for 2 min, with a final extension of 10 min at 72 °C. Full length amplified gene product was cloned in pGEM-T easy vector, as per manufacturer’s instructions. Mutations were confirmed by sequencing, and all the genes (WT and variants) were submitted to gene bank. Following confirmation of the sequence, genes encoding WT and variants were sub-cloned in pQE-30 UA expression vector and transformed in *Escherichia coli* M15 cells. Transformed cells were selected on LB agar medium plates containing ampicillin (100 μg) and kanamycin (35 μg).

### Expression and purification of recombinant protein

*E. coli* M15 cells harboring recombinant pQE-30 UA plasmids were cultivated overnight at 37 °C in 5 ml LB media having antibiotics as mentioned above. Next day, 1 % overnight grown culture was inoculated into 500 ml media and the expression was induced by addition of 1 mM IPTG when OD was reached ~0.4–0.6. Cells were harvested after 6 h growth and the extracellularily secreted protein (WT and variants) was purified from the supernatant as reported previously (Sharma et al. 2012a). Recombinant protein samples from WT and variants were purified separately at 4 °C, unless and otherwise stated.

### Enzyme assay

Enzyme assays were carried out according to the Sigurgisladottir et al. (1993). Phosphate buffer 0.8 ml (0.05 M, pH 8.0) was premixed with 0.1 ml enzyme (appropriately diluted) and 0.1 ml of 0.002 M *p*-nitrophenyl laurate. The reaction mixture was incubated at 50 °C for 10 min. Reaction was stopped by adding 0.1 M Na_2_CO_3_ (0.25 ml). Reaction mixture was centrifuged and supernatant was used to determine the enzyme activity. Enzyme activity was measured at 420 nm in UV/Vis spectrophotometer (JENWAY 6505, UK). One unit of enzyme activity is defined as the amount of enzyme, which liberates 1 μmole of *p*-nitrophenol from pNP-laurate as substrate/min under standard assay conditions. The total enzyme activity was expressed in U/ml, whereas the specific activity was expressed as U/mg of protein.

### Effect of temperature on enzyme activity and stability

To calculate temperature optima, the purified lipase proteins (WT and variants) were assayed at different temperatures (20–80 °C). To study the thermal inactivation, the enzymes were incubated at different temperatures (20–80 °C) for 30 min. After heat treatment the reaction tubes were kept in ice for 15 min and assayed for enzyme activity. Enzyme without incubation was taken as control (100 %). Similarly, the thermal inactivation was also studied at 55 and 60 °C for different time intervals. The enzyme activity at the start of the experiment was taken as 100 %, and the residual lipase activity after incubation was determined. Reaction mix without enzyme served as blank.

### Effect of pH on enzyme activity and stability

Optimum pH for the purified lipase (WT and variants) was determined by assaying these enzymes at various pH i.e. sodium acetate—pH 5.0, sodium phosphate—pH 6.0–8.0, Tris.HCl—pH 9.0, Glycine NaOH—pH 10.0–11.0, at 50 °C and 40 °C respectively. The pH stability assays of the lipases were performed by pre-incubating these enzymes in presence of 0.05 M buffer of different pH (5.0–11.0) for 1 h at room temperature, followed by enzyme assay.

### Specificity

Substrate specificity for WT and its variants were determined using pNP ester (final concentration 0.2 mM) of following chain lengths: pNP-acetate (C_3_), pNP-butyrate (C_4_), pNP-caprylate (C_8_), pNP-deconate (C_10_), pNP-laurate (C_12_), pNP-myristate (C_14_), pNP-palmitate (C_16_), pNP stearate (C_18_) from Sigma (USA) were dissolved in absolute alcohol, and used in enzyme assay reaction according to standard assay method.

### Kinetic study

Enzyme activity of WT and its variants were determined as a function of range of substrate concentration **(**0.01–2.5 mM of pNP laurate). The Michaelis–Menten constant (*K*_m_) and maximum velocity for the reaction (*V*_max_) with pNP-laurate as substrate were calculated by Lineweaver–Burk plot. The *k*_cat_ and *k*_cat_/*K*_m_ were also calculated and the results were compared.

### Gene submission

All genes (WT and variants) were submitted in gene bank (NCBI) with accession numbers WT-FJ392756.1, lip M1-GU292533, lip M2-GU292534, and lip M3**-**GU292535.

### Molecular modeling

We employed MODELLER 9v7 program to build the homology models, using highly identical templates, for which crystal structure was reported in Protein Data Bank (PDB). The crystal structure of *Bacillus stearothermophilus* L1 lipase (PDB ID: 1KU0, sequence identity 96 %) (Jeong et al. 2002) and *Geobacillus thermocatenulatus* (PDB ID: 2W22, sequence identity 94 %) (Carrasco-López et al. 2009) showed high structural identity, when BLASTP (Basic Local Alignment Search Tool for Protein at NCBI) was performed. Furthermore, *Bacillus stearothermophilus* L1 lipase (PDB ID: 1KU0) was used as template for construction of a homology models, and all 3-D models of the lipase (WT and variants) were built, starting from the template. Energy minimizations of the modeled structures were carried out using forces field GROMOS96 43a1 and steepest descent method for 1,000 steps in the GROMACS 3.3.1 (Sali et al. 1995; Lindahl et al. 2001). Additionally, the loop regions (Leu314-Asn321 and Thr353-His358) were also modeled using loop script in MODELLER 9v7 and eventually verified and validated on Structure Analysis and Verification Server (http://nihserver.mbi.ucla.edu/SAVES). The final loop refinement structures were used for structural comparison analysis. Additionally, *Eris* server was used to calculate the changes in protein stability induced by mutations (ΔΔG) utilizing the recently developed Medusa modeling suite. The server is freely accessible online (http://eris.dokhlab.org) (if ΔΔG <0: stabilizing mutations ΔΔG >0: destabilizing mutations) (Ding and Dokholyan 2007).

## Results and discussion

**Fig. 1:**
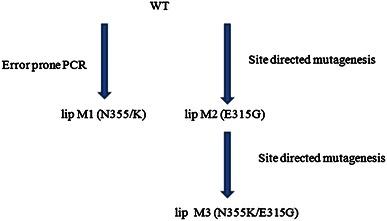
Schematic presentation for the generation of variants

Protein engineering method offers valuable tools to modify various properties of the biocatalysts that include e.g. the operational stability under denaturing conditions, enantioselectivity and chain length specificity etc. (Fujii et al. 2005; Magnusson et al. 2005; Reetz 2000; Koga et al. 2003). Here, we created mutant lip M2 and lip M3 from a native (WT) lipase, cloned from a metagenomic DNA extracted and purified from hot spring soil (Sharma et al. 2007), as depicted in Fig. 1. The results were compared with lip M1 and WT.

### Protein expression, purification and Biochemical properties

**Fig. 2:**
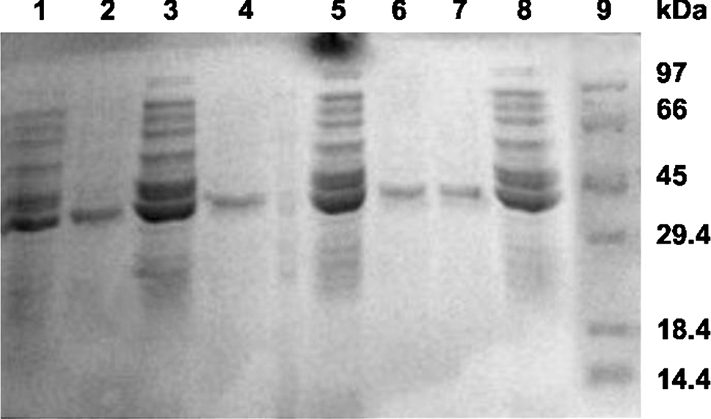
SDS-PAGE analysis for wild type and mutant lipase. *Lane 1, 3, 5, 8*: induced culture from WT and variants. *Lane 2, 4, 6, 7*: purified protein sample from WT and variants after Phenyl-Sepharose chromatography (*Lane 1, 2* (WT), *3, 4* (lip M1) *5, 6* (lip M2) *7, 8* (lip M3). *Lane 9*: protein molecular weight marker

**Fig. 3:**
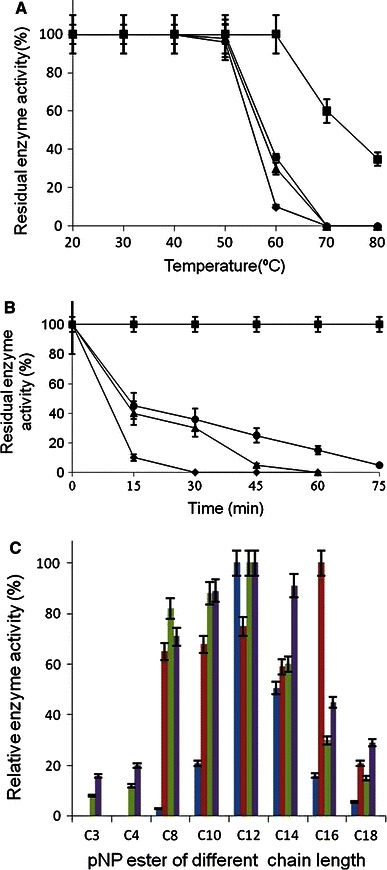
**a** Thermostability assay for WT and variant proteins at different temperature performed by pre-incubating all enzymes at different temperature (20–80 °C) for 30 min followed by cooling of enzymes for 10 min on ice prior to enzyme assay. (*Filled square*) lip M1, (*filled circle*) lip M3, (*filled triangle*) WT, (*filled diamond*) lip M2. **b** Thermal denaturation assays for the WT and variant proteins at 55 °C for varying time periods, (*filled square*) lip M1, (*filled circle*) lip M3, (*filled triangle*) WT, (*filled diamond*) lip M2. **c** Substrate specificity of the WT and variant proteins (*blue*) WT, (*brown*) lip M1, (*purple*) lip M2, (*green*) lip M3

Protein purified from WT and its variants showed single band of expected molecular weight on 12 % SDS-PAGE, as deduced from number of amino acids present in the mature polypeptide (Fig. 2). We further compared purification profile of WT and enzyme variants (data not shown**)** and observed that variant lip M1 had highest specific enzyme activity i.e. 3,090 ± 14 U/mg compared to the WT, lip M2 and lip M3 whose specific enzyme activity was calculated to be 2,022 ± 31, 1,816.2 ± 18 and 1,972 ± 50 U/mg protein respectively. All enzymes demonstrated virtually a comparable activity and stability over wide range of pH (data not shown). Furthermore, all of them had displayed enzyme activity over broad range of temperature, with optimum enzyme activity observed at 50 °C, except to the lip M1 that displayed optimum enzyme activity at 40 °C (data not shown). Additionally, the thermostability of WT and variants tested over a range of temperature i.e. 20–80 °C for 30 min did not show loss in the enzyme activity until 50 °C. However at 60 °C, all of them showed a decrease in enzyme activity, apart from lip M1 (Fig. 3a). Thermal stability assays were also performed at 55 °C for varying time points (Fig. 3b). Various thermostability assays revealed following order for the thermal denaturation i.e. lip M1 > lip M3 > WT > lip M2. In addition, half life of all the proteins was also calculated and compared at 60 °C (Table 1).

**Table 1:** Comparative biochemical studies for the WT and variant enzymes

Biochemical properties	WT	Lip M1	Lip M2	Lip M3
Specific activity	2,022 ± 31	3,090 ± 14	1,816 ± 18	1,972 ± 50
Temperature optimum (°C)	50	40	50	50
pH optimum	9	9	7–8	9
Half life at 60 °C	5 min	14 h	<5 min	>5 min
pH stability	8–9	7–9	7–8	8–9
*k*_m_ (μM)	0.73	0.33	1.33	1.18
*V*_max_ (μmol/ml/min)	239 ± 16	312	28	23
*k*_cat_ (s^−1^)	569	5,199	249	189
*k*_cat_*/K*_m_ (μM^−1^ s^−1^)	779	15,754	187	160
Preferred substrate	pNP-laurate	pNP-palmitate	pNP-laurate	pNP-laurate

### Biochemical kinetics study

Kinetic parameters determined for the WT and variants using *p*-nitrophenyl laurate as substrate, demonstrated great variation (supplementary figures, S1–S4). The mutant lip M1 displayed *k*_cat_ ~9 times higher than WT enzyme, whereas lip M2 and lip M3 showed ~2 fold less *k*_cat_. The overall catalytic efficiency i.e. *k*_cat_/*K*_m_ of lip M1 showed ~20 fold increase, whereas lip M2 and lip M3 illustrated ~4 fold decrease in the catalytic efficiency than the WT, which is ~85 and ~99 folds lower than lip M1 respectively (Table 1). Information gathered from the biochemical kinetics data suggested that mutation near the active site might affect the binding of the substrate to the active site, and may be attributed to more flexibility and distortion in the structure on bringing in E315G mutation. All over it appears that the mutation has either affected the binding of the substrate molecule in the catalytic site or resulted in dispersion of the enzymatic product away from the micro-catalytic environment.

### Effect of p-NP ester chain on enzyme activity

Substrate specificity of the WT and variants was evaluated and compared by testing enzyme activity in presence of pNP-esters of varying carbon chain length C_3_–C_18_ (Fig. 3c). It is evident from the figure that WT, lip M2 and lip M3 displayed maximum enzyme activity with pNP-laurate while lip M1 showed maximum activity towards C_16_ (pNP–palmitate). In addition, lip M2 and lip M3 also demonstrated lipase activity towards short chain pNP- esters. Alteration of the specificity may be the result of changed conformation that has affected the catalytic pocket of the enzyme.

### Structural implications and molecular modeling

**Fig. 4:**
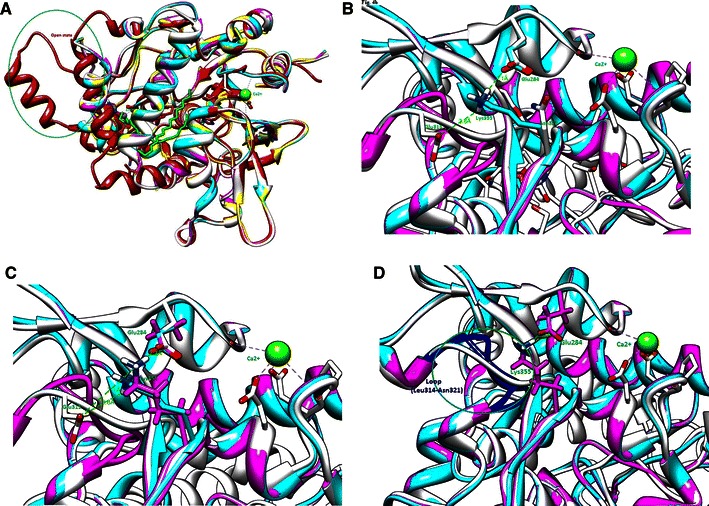
Modeled structures of WT (*white color*) and mutants lipases (*cyan* and *magenta color*), superimposed on crystal structure 1KU0 (*maroon color*) in various color representation depicted in **a**; modeled structure of variant N355K (*cyan color*) showing H-bonding between Lys355 and Glu284 with a distance, 2.1 Å (Lys355 NH–O=C Glu284) and between Lys355 and Glu3153.8 Å (Lys355 NH–O=C Glu315) depicted in **b**; double mutated model (*magenta color*) showing H-bonding interaction between Lys355 and Glu284 with distance 2.1 Å (Lys355 NH–O=C Glu284), superimposed on mutant N355K showing distance 7.1 Å (Lys355 NH–O=C Glu315) with WT amino acid Glu315, as depicted in **c**; the overall structural changes in loop region (*blue color*) of double mutated and WT form, after loop refinements shown in **d**

Mutation near the active site residues can influence both hydrogen bonding and conformational stability (Offman et al. 2011; Morley and Kazlauskas 2005). Previous studies also established that mutation altering enzyme structure and functions exists at the vicinity of the active site (Takase 1993; Ollis et al. 1992). In the present investigation, we report that less thermo stability and catalytic efficiency of lip M2 and lip M3 by E315G mutation may be attributed to its presence at vicinity of the active site. Substituting a negatively charged polar amino acid (glutamic acid) to a non polar and flexible amino acid (glycine) in mutant lip M2 and lip M3 may affect the conformational plasticity of the enzyme. In general, glycine is reported to be present in those part of the protein structures which are forbidden to other amino acids, while glutamic acid commonly exist on the protein surface, and can interact with other amino acids. Furthermore, in order to demonstrate the effect of these mutations at three dimensional structural levels, we performed three dimensional modellings. To verify models, they were energy minimized using GROMACS 3.3.1 program and further validated by Ramachandran plot. The plot revealed presence of ~91.1 % amino acids in the allowed core region, while ~8.3 % were located in the additionally allowed region, and no residues were observed in the disallowed region, hence illustrated their best fit. Further, all these models exhibited superimposed view of the template IKU0, and hence high structural similarity (rmsd values for the model struture was found to be, C-α 0.181 with reference IKU0 and C-α 4.76 with reference 2W22), except in the loop region Leu314-Asn321 (Fig. 4a). In addition, all of them exhibited typical α/β hydrolase fold, a characteristics feature of lipases with conserved metal ion binding sites (Zn^2+^, Ca^2+^). Further refining of the structural models in loop regions Leu314–Asn321 and Thr353–His358 showed structural flexibility in Leu314–Asn321, owing to replacement of glutamic acid with glycine in lip M2 mutant. On the other side, mutant N355K (lip M1) demonstrated side chain conformational change in the loop region Thr353–His358 and resulted in extensive H-bonding of Lys 355 with Glu284 that renders the protein more thermostable as mentioned in our previous study (Sharma et al. 2012b). Computational molecular modeling experiment further demonstrated compact packing of positively charged amino acid lysine in between two negatively charged amino acids Glu284 and Glu315 and the distance measured was predicted to be 2.1 and 3.8 Å respectively (Fig. 4b), which may be another rationale for the improved protein thermostability in lip M1. Consequently, when both these mutations were brought together (lip M3), mutation E315G resulted in disruption of the electrostatic interactions between Glu315 and Lys355. To know whether this altered structural plasticity (conformation) has affected the distance between Lys355 and Glu284, and Lys355 and Glu315, we superimposed lip M3 modeled structure on WT model. In fact, we observed an increase in the distance between Lys355 and Glu284 (2.1–2.2 Å) and Lys355 and Glu315 (3.8–7.1 Å) in lip M3 (Fig. 4c) which may be accredited to augmented flexibility in the loop region Leu314–Asn321 (Fig. 4d) as predicted. Our observations were further strengthened by calculating change in protein stability induced by mutations (ΔΔG). The values obtained for different proteins were as follow e.g. E315G showed +4.39, for N355K +1.02, whereas dual mutant (G315E/N355K) it was −4.87, respectively (Ding and Dokholyan 2007). Therefore, our present investigation provide strong evidence that several loop conformational changes accompanied by distortion in electrostatic interactions resulted in loss of protein thermo stability and enzyme activity in the lip M3.

## Conclusion

Altogether, our biochemical, circular dichroism and computational studies provide strong evidence that an amino acid substitution at positions 355 is critical for lipase stability and activity, whereas, residue at position 315 had only a marginal effect on its own. The mutation at 315 was able to nullify the enhanced thermostability and catalytic efficiency acquired by mutant N355K, might be due to altered loop conformations and disruption of the electrostatic interactions. We strongly feel that data in the manuscript can contribute in understanding the structural and functional of proteins.

## Electronic supplementary material

Below is the link to the electronic supplementary material.

Supplementary material 1 (JPEG 45 kb)

Supplementary material 2 (JPEG 43 kb)

Supplementary material 3 (JPEG 72 kb)

Supplementary material 4 (JPEG 58 kb)
