# Supplementary figures and images for: Insights into controlling role of substitution mutation, E315G on thermostability of a lipase cloned from metagenome of hot spring soil
Source: 3 Biotech. 2013 Jun 2;4(2):189–96. doi: 10.1007/s13205-013-0142-4 (PMC3964248; doi:10.1007/s13205-013-0142-4)

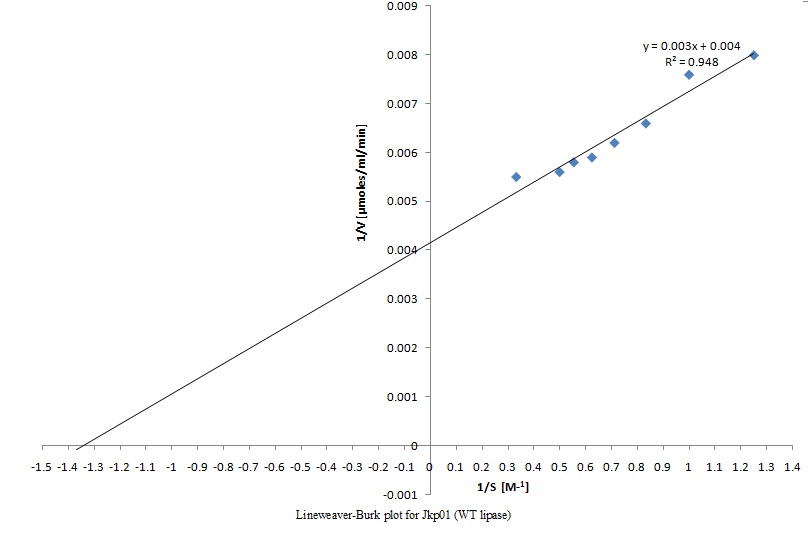

Supplement: Supplementary file 1 — Supplementary material 1 (JPEG 45 kb) [file 13205_2013_142_MOESM1_ESM.jpg]

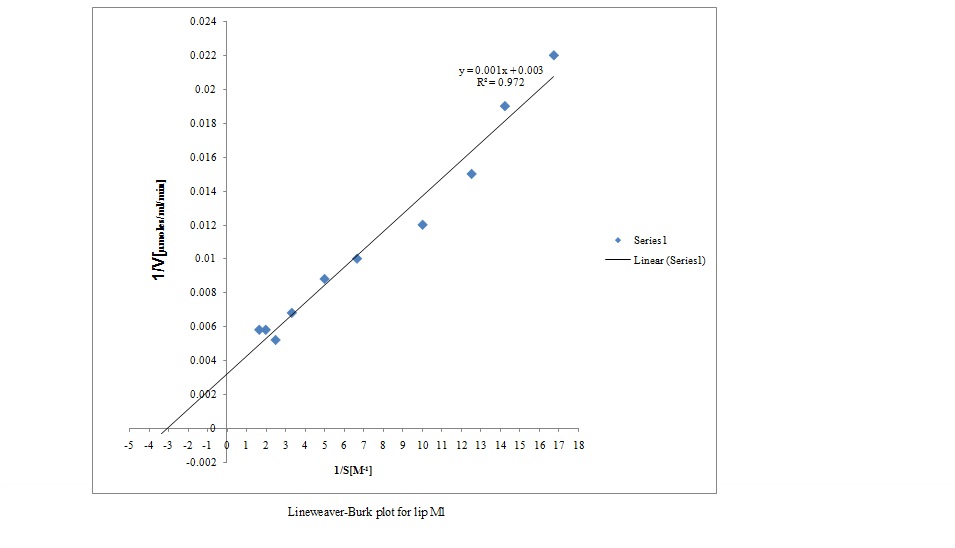

Supplement: Supplementary file 2 — Supplementary material 2 (JPEG 43 kb) [file 13205_2013_142_MOESM2_ESM.jpg]

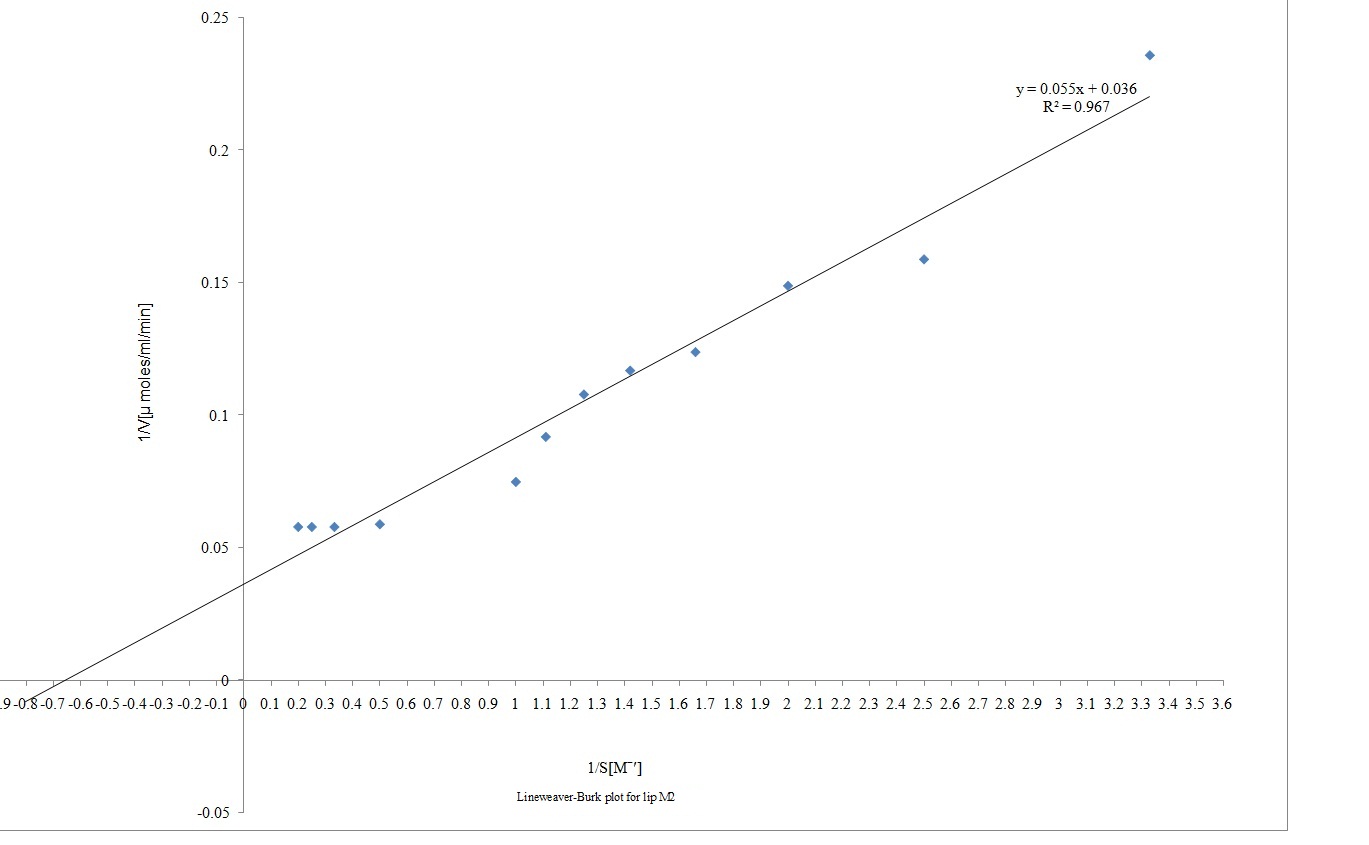

Supplement: Supplementary file 3 — Supplementary material 3 (JPEG 72 kb) [file 13205_2013_142_MOESM3_ESM.jpg]

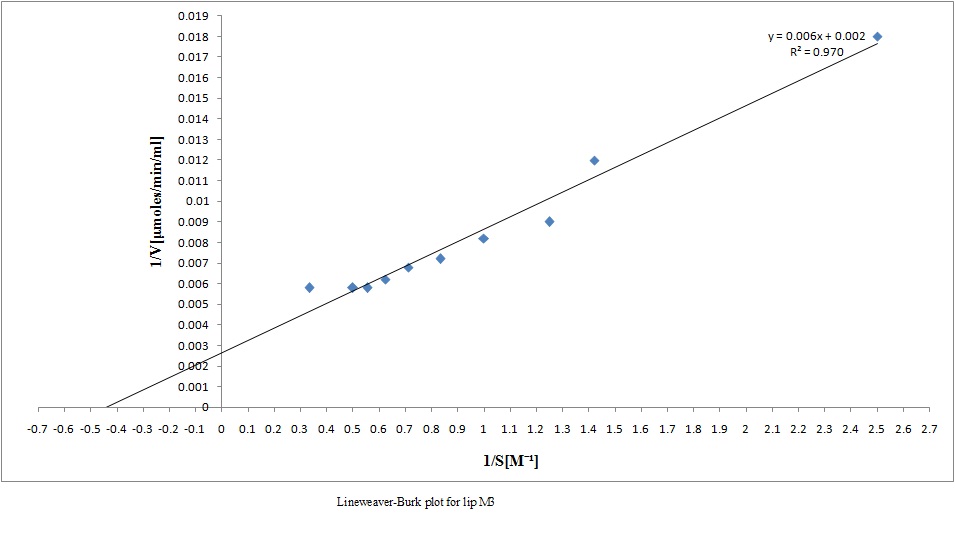

Supplement: Supplementary file 4 — Supplementary material 4 (JPEG 58 kb) [file 13205_2013_142_MOESM4_ESM.jpg]
